# Impact of Gut Microbiome on Hypertensive Patients With Low-Salt Intake: Shika Study Results

**DOI:** 10.3389/fmed.2020.00475

**Published:** 2020-09-02

**Authors:** Satoshi Nagase, Shigehiro Karashima, Hiromasa Tsujiguchi, Hirohito Tsuboi, Sakae Miyagi, Mitsuhiro Kometani, Daisuke Aono, Takuya Higashitani, Masashi Demura, Hiroyuki Sakakibara, Akihiro Yoshida, Akinori Hara, Hiroyuki Nakamura, Yoshiyu Takeda, Hidetaka Nambo, Takashi Yoneda, Shigefumi Okamoto

**Affiliations:** ^1^Department of Laboratory Science, Faculty of Health Sciences, Kanazawa University, Kanazawa, Japan; ^2^Department of Endocrinology and Metabolism, Kanazawa University Hospital, Kanazawa, Japan; ^3^Department of Environmental and Preventive Medicine, Advanced Preventive Medical Sciences, Kanazawa University, Kanazawa, Japan; ^4^Division of Psychosomatic Medicine, Institute of Medical, Pharmaceutical, and Health Sciences, Kanazawa University, Kanazawa, Japan; ^5^Department of Hygiene, Kanazawa University Graduate School of Medicine, Kanazawa, Japan; ^6^Graduate School of Agriculture, University of Miyazaki, Miyazaki, Japan; ^7^Department of Oral Microbiology, Matsumoto Dental University Graduate School of Oral Medicine, Shiojiri, Japan; ^8^School of Electrical, Information, and Communication Engineering, College of Science and Engineering, Kanazawa University, Kanazawa, Japan; ^9^Department of Health Promotion and Medicine of the Future, Kanazawa University Graduate School of Medicine, Kanazawa, Japan

**Keywords:** gut microbiome, blood pressure, salt-intake, renin-angiotensin-aldosterone system, hypertension

## Abstract

Salt intake is one of the most important environmental factors impacting hypertension onset. Meanwhile, the potential roles of the gut microbiome (GM) in altering the health status of hosts have drawn considerable attention. Here, we aimed to perform an observational study to investigate the impact of intestinal bacterial flora in hypertensive patients with low-salt or high-salt intake. A total of 239 participants were enrolled, and their gut microbiomes, clinical and demographic details, as well as physiological parameters pertaining to the renin-angiotensin-aldosterone system and inflammatory cytokine profiles, were examined. The participants were classified into four groups based on the presence of different enterotype bacteria, as determined via cluster analysis, and salt intake: low salt/GM enterotype 1, low salt/GM enterotype 2, high salt/GM enterotype 1, and high salt/GM enterotype 2. Results show that the prevalence of hypertension was significantly lower in the low-salt/GM enterotype 2 group (27%) compared to the low salt/GM enterotype 1 group (47%; *p* = 0.04). Alternatively, no significant differences were observed in hypertension prevalence between the two high-salt intake groups (GM enterotype 1 = 50%, GM enterotype 2 = 47%; *p* = 0.83). Furthermore, The low-salt/GM enterotype 2 was higher in the relative abundances of *Blautia, Bifidobacterium, Escherichia-Shigella, Lachnoclostridium*, and *Clostridium sensu stricto than* the low-salt/GM enterotype 1. differed significantly between the GM enterotypes. These results suggested that consumption of a low-salt diet was ineffective in regulating hypertension in individuals with a specific gut bacteria composition. Our findings support the restoration of GM homeostasis as a new strategy for controlling blood pressure and preventing the development of hypertension.

## Introduction

Hypertension has become an important global health issue and is a major risk factor for cardiovascular, cerebrovascular, and kidney diseases (1, 2). It is believed that the etiology of hypertension depends on the complex interplay of both genetic and environmental factors (3, 4). Salt intake is one of the most important environmental factors of hypertension onset. For instance, the Intersalt Cooperative Research Group found significant positive relationships between 24 h urinary sodium excretion and blood pressure (BP) in the study participants (5). In addition, the Dietary Approaches to Stop Hypertension (DASH) interventional study showed that dietary salt-intake patterns may affect BP in the adult population with BP in the high normal range compared to those that are moderately hypertensive (6). Most studies show that excess sodium consumption raises BP in a dose-dependent manner; however, salt sensitivity, that is how BP responds to salt, varies, with less than one-third of normotensive individuals and less than one-half of hypertensive individuals classified as salt sensitive (7–10). Known sources of such variability include genetic polymorphisms of the associated renin-angiotensin-aldosterone system (RAAS), dietary intake, and kidney disease.

In recent decades, the potential roles of the gut microbiome (GM) in altering the health status of hosts have drawn considerable attention. Several lines of evidence suggest a link between GM and lifestyle disease, including diabetes, obesity, and hypertension (11–13). For instance, GM dysbiosis accompanies hypertension in rodents (14, 15). In Dahl rats, distinct differences in metagenomic composition have been identified for salt-sensitive and salt-resistant strains (16). Furthermore, the GM of salt-sensitive rats is suggested to have symbiotic relationships with their hosts (16). This suggests that changes in GM precede the onset of hypertension, which is supported by the findings of Wilck et al., who demonstrated that feeding mice a high-salt diet decreases the proportion of gut *Lactobacillus murinus*, which is associated with increased number and activation of TH17 cells (17). These cells secrete a pro-inflammatory cytokine, interleukin-17 (IL-17), which is believed to promote high BP and accompanying inflammation in artery walls (17). However, most of these studies were performed using animal models, which may not directly translate to human disease. Furthermore, there are only limited human clinical trials that have been performed to decipher the relationships between dietary salt, GM, immunological reactions, and BP. The aim of the current observational study was to, therefore, investigate the impact of the intestinal bacterial flora on hypertensive patients with low-salt intake.

## Materials and Methods

### Study Population

We used cross-sectional data of the Shika study, which is a population-based study that aims to establish a method to prevent lifestyle-related diseases (18, 19). It includes interviews, questionnaires, and health examinations. Health examination data was collected between March 2014 and January 2018 from the residents of Shika, a town with more than 20,000 residents (20), located in a north area of Ishikawa Prefecture, Japan (21). The present study was conducted from December 2017 through January 2018 with four model districts in Shika being selected, including Horimatsu, Higashi-Masuho, Tsuchida, and Togi.

### Ethical Considerations

The study was approved by the Ethics Committee for Human Studies at Kanazawa University Hospital (No. 1491) and was performed in accordance with the principles of the Declaration of Helsinki and the Microorganism Safety Management Regulations of Kanazawa University. All participants were provided an explanation of the research and subsequently provided written informed consent prior to the collection of gut microorganisms. Collected microorganisms were processed in a biosafety level-2 laboratory.

### Data Collection

Data through the Shika study were collected by participant interviews and included demographics, such as age, sex, underlying diseases, and medications. Height, weight, and BP were measured during study visits. Specifically, BP was measured when the subjects were seated in a chair. A suitably sized cuff was placed on the right upper arm and attached to UM-15P (Parama-tech Co., Ltd., Fukuoka, Japan) and HEM-907 (OMRON Co., Ltd., Kyoto, Japan). BP monitors contained automated digital sphygmomanometer based on the oscillometric method (18). Hypertension was defined as sBP of 140 mmHg, dBP of 90 mmHg, or if participants reported use of antihypertensive drugs. Venous blood samples were collected in the mornings after 15 min periods of rest following a 12 h overnight fast.

Daily salt-intake was evaluated by urine sodium levels and creatine ratios in urine samples (22). Estimated glomerular filtration rates (eGFR) were calculated using serum creatinine levels. Plasma renin activity (PRA) and plasma aldosterone concentration (PAC) were measured using radioimmunoassays, as previously reported (23). Serum levels of cytokines, including, granulocyte-macrophage colony-stimulating factor (GM-CSF), interleukin-17a (IL-17a), and tumor necrosis factor alfa (TNF-α) were measured using a MILLIPLEX MAP Human High Sensitivity T Cell Panel-Immunology Multiplex Assay (Merck KGaA, Darmstadt, Germany) and Luminex® 200TM flow cytometry system (Thermo Fisher Scientific, MA, US).

### Stool Sample Collection and DNA Extraction

Stool samples were collected from 254 participants. However, 15 of the samples were excluded due to the subjects taking antimicrobial or steroid drugs, resulting in 239 stool samples for analysis. Fecal sample collection was accomplished using clean paper (AS ONE Inc., Osaka Japan) and a clean spatula with plastic tube (AS ONE Inc.). The fecal samples were then transferred to sterile closed plastic tubes the in morning and transported to the laboratory on ice within the day. The samples were stored at −80°C until DNA extraction. Whole DNA was extracted from the fecal samples using NucleoSpin® DNA Stool (Macherey-Nagel Inc., Düren Germany) according to the manufacturer's instructions.

### Next Generation Sequencing (NGS)

The extracted DNA of gut microbiome was processed for 16S rRNA gene sequencing by NGS using methods previously described (24). V3-4 regions of the 16S rRNA gene were amplified using Ex Taq® Hot Start Version polymerase and TaKaRa PCR Thermal Cycler Dice® Gradient (TaKaRa Bio Inc., Shiga, Japan). The PCR products were purified by Agencourt AMPure XP magnetic beads (Beckman Coulter, Inc., CA, USA). The amount of the PCR products were measured by Qubit® dsDNA HS Assay Kit and Qubit® 3.0 fluorometer (Thermo Fisher Scientific, Inc.). All purified PCR products were sent to Hokkaido System Science Co., Ltd. (Hokkaido, Japan) for Illumina MiSeq sequencing. The NGS data were registered in the DNA Data Bank of Japan (DDBJ; accession number PRJDB8820).

### Microbiome Analysis

Microbiome analysis was performed according to methods reported in a previous study (24). The pair-end sequences were filtered by Sickle (version 1.33) (25) and assembled by PANDAseq (version 2.11) (26). Removed chimera sequences were used by USEARCH (version 10.0.240_i86linux32) (27) and Silva 16S rRNA database (release 132) (28). From non-chimeric sequences, the “pick_de_novo_otus.py” command in Qiime (version 1.9.1) and the Silva 16S rRNA gene database (release 132) was used to generate operational taxonomic unit (OTU) (97% similarity threshold) (29). Finally, global singletons were removed using the “filter_otus_from_otu_table.py” command in Qiime.

### Statistics

R Package (version 3.5.0) software was used for all statistical analyses (30). Box plots showed the median, 1st quartile, 3rd quartile with 1st quartile + 1.5 × interquartile range (IQR), and 3rd quartile – 1.5 × IQR whiskers, and points exhibited outliers. Participant characteristics and relative abundance among each group were compared using one-way analysis of variance (ANOVA) and analysis of covariance (ANCOVA) with adjustment for age, sex, and body mass index (BMI). The relative abundance of each microgram was compared using the DESeq2 package (31). Cluster analysis was performed based on the previous study (32). Principal component analysis (PCA) used Jensen-Shannon divergence values based on the genus level. Cluster analysis was performed used Partitioning Around Medoid (PAM) clustering. The PCA and cluster analysis were performed using “philentropy” and “pamr” package (32, 33). A random forest analysis used the randomForest package of R (34). All microorganisms and participant information [age, sex, BMI, PRA, PAC, eGFR, diabetes mellitus (DM) rate, hyperlipidemia (HL) rate, IL-17a levels, GM-CSF levels, and TNF-α levels] were evaluated with the random forest function using default parameters. Random forest analysis of bacterial genera and participant information contained out of bag error rates of 44.3 and 37.7%, respectively. The similarity of microbiome composition between each participant group was assessed by permutational multivariate analysis of variance (PERMANOVA) using the “adonis” command in the vegan package of R (10,000 simulations) (35).

## Results

### Enterotype Clustering and Salt Intake

In total, 239 participants were included for analysis. The prevalence of hypertension was 44.8% in the study population. Mean systolic BP (SBP) and diastolic BP (DBP) were 136 ± 17 and 80 ± 11 mmHg, respectively. The mean daily salt-intake was 9.4 ± 1.9 g/day (median 9.6 g/day). The antihypertensive drug usage rate was 11.7% (28/239). Specifically, 25 participants were treated with angiotensin converting enzyme inhibitors and/or angiotensin receptor blockers. There was no participant treated with mineralocorticoid receptor antagonists. There were three patients treated with the other antihypertensives excluding RAAS inhibitors.

**Figure 1:**
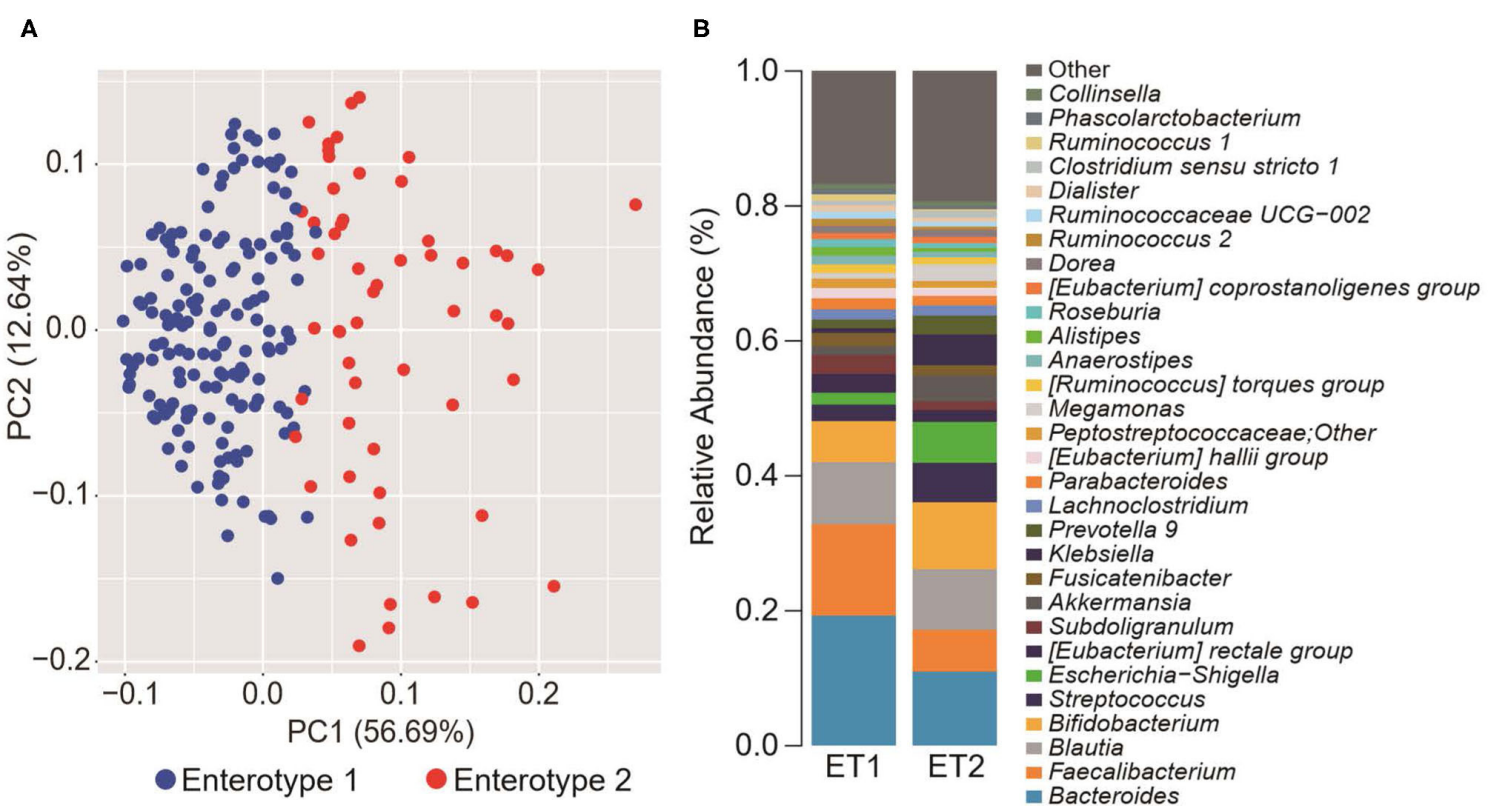
Gut microbiome differences determined by clustering analysis. **(A)** Principal component analysis (PCA) plot generated using the Partitioning Around Medoid (PAM) clustering method based on Jensen-Shannon distance of operational taxonomic unit (OTU) counts. Blue, enterotype 1; Red, enterotype 2. **(B)** Relative abundance of the top 30 microorganisms identified by the gut microbiome cluster.

To explore the potential differences between hypertension rates with respect to microbiome and salt intake, the participants were separated into four groups based on cluster analysis. First, the GMs of the participants were structured into two enterotypes using the PAM clustering method based on Jensen-Shannon distance (Figures 1A,B). There was no significant difference in the participant information between the two enterotypes (Supplemental Table 1). Next, two groups were formed based on median salt-intake values, a high-salt group which was defined as higher than 9.6 g/day of daily salt-intake, and a low-salt group which was defined as the lower. There was no significant difference in relative microgram levels of salt between the high-salt and low-salt groups. Finally, we classified the study participants into four groups based on GM enterotype and salt intake. These included a low salt/GM enterotype 1 group, a low salt/GM enterotype 2 group, a high salt/GM enterotype 1 group, and a high salt/GM enterotype 2 group.

### Clinical Background

The clinical background information for the four groups of participants based on salt intake and GM are shown in Table 1. There were significant differences among the four groups with respect to the prevalence of females (*p* = 0.04), daily salt intake (*p* < 0.01), and PAC (*p* < 0.01). However, no differences were observed for any other clinical parameters including sBP (*p* = 0.21) and dBP (*p* = 0.34). There were, however, significant difference observed between the low-salt groups with respect to the prevalence of females (*p* = 0.02). Meanwhile, the high-salt groups did not exhibit significant differences between enterotype 1 and enterotype 2 (Supplemental Table 2).

**Table 1:** Characteristics of the four groups of study participants categorized by gut microbiome clustering and daily salt-intake.

**Characteristics**	**All**	**Low salt intake**		**High salt intake**	
	**(*n* = 239)**	**Enterotype 1 (*n* = 83)**	**Enterotype 2 (*n* = 37)**	**Enterotype 1 (*n* = 89)**	**Enterotype 2 (*n* = 30)**
Age, y	63 ± 10	64 ± 11	61 ± 11	63 ± 9	63 ± 9
Female, %^*^	52.3	66.3^‡^	40.5	47.2	43.3
BMI, kg/m^2^	23.3 ± 3.1	23.0 ± 3.7	22.6 ± 2.7	23.7 ± 2.5	23.4 ± 3.3
Hypertension, %	44.8	47.0^‡^	27.0	49.4	46.7
SBP, mmHg	136 ± 17	135 ± 19	132 ± 17	139 ± 17	138 ± 17
DSP, mmHg	80 ± 11	78 ± 12	78 ± 9	82 ± 10	83 ± 11
Salt intake, g/day^†^	9.4 ± 1.9	7.9 ± 1.2	7.8 ± 1.3	10.9 ± 1.2	10.8 ± 1.1
eGFR, mL/min/1.73 m^2^	68.4 ± 12.1	66.0 ± 12.3	67.5 ± 11.5	69.6 ± 11.1	72.5 ± 12.1
PRA, ng/mL/h	2.4 ± 5.6	2.8 ± 8.4	3.0 ± 5.4	1.9 ± 2.0	1.8 ± 2.2
PAC, pg/mL^†^	140.6 ± 69.2	155.0 ± 86.9	161.5 ± 57.9	124.5 ± 51.9	122.3 ± 54.1
GM-CSF, pg/mL	8.1 ± 12.6	9.3 ± 15	5.8 ± 4.1	8.9 ± 14.0	5.3 ± 12.6
IL17a, pg/mL	2.0 ± 1.3	2.1 ± 1.2	2.0 ± 1.1	2.1 ± 1.5	1.7 ± 1.3
TNF-α, pg/mL	1.5 ± 0.6	1.5 ± 0.6	1.4 ± 0.4	1.6 ± 0.8	1.3 ± 0.6
Antihypertensive %	11.7	14.5	18.9	5.6	13.3

* P < 0.05 and

† *P < 0.01 based on analysis of covariance in four groups*.

‡ *P < 0.05 vs. enterotype 2 (ANCOVA).: BMI, body mass index; SBP, systolic blood pressure; DBP, diastolic blood pressure; eGFR, estimated glomerular filtration rate; PAC, plasma aldosterone concentration; PRA, plasma renin activity; GM-CSF, granulocyte-macrophage colony-stimulating factor; IL-17a, interleukin-17a; TNF-α, tumor necrosis factor alpha*.

### Prevalence of Hypertension a Random Forest Analysis

**Figure 2:**
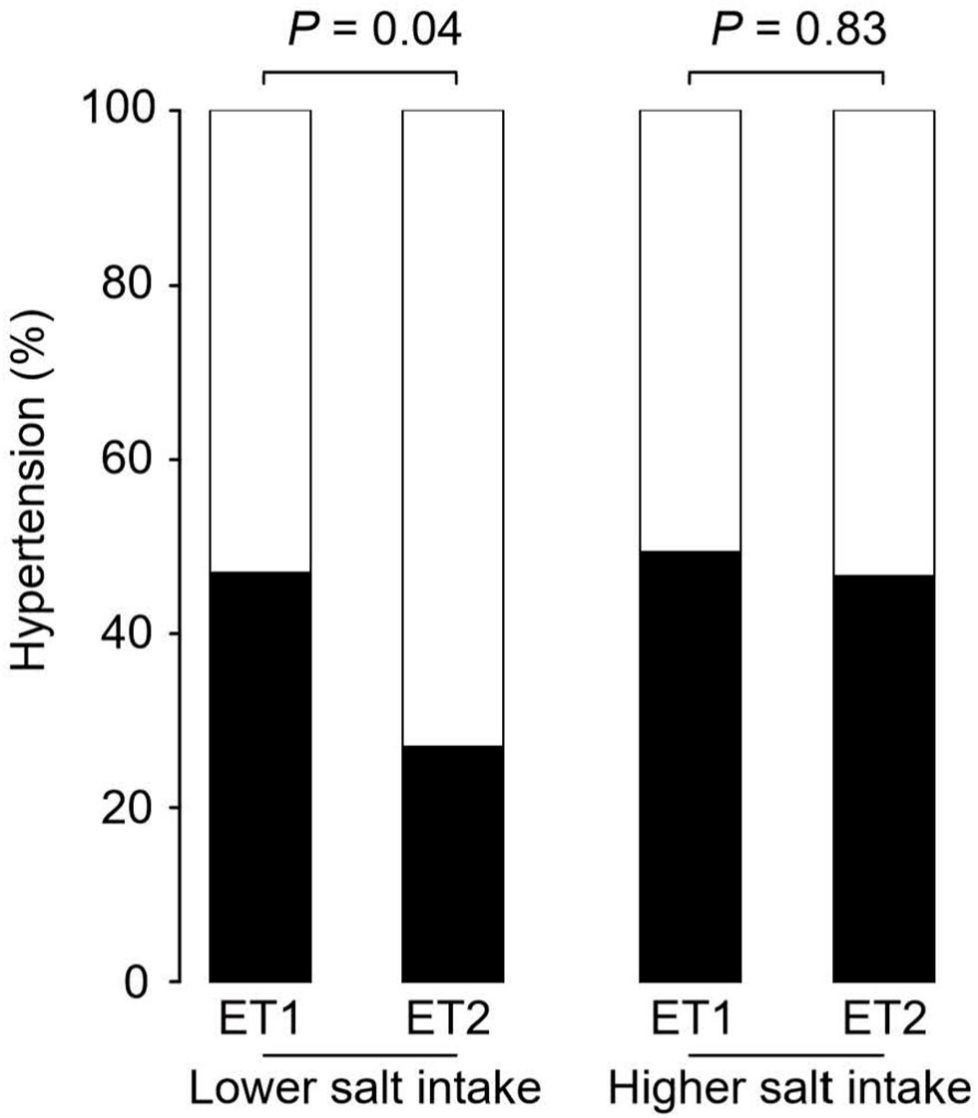
Hypertension prevalence rates for enterotype and salt-intake groups. Hypertension rates are shown based on GM clustering and salt-intake. Black, hypertension rate; White, non-hypertension rate. *P*-values were calculated using Fisher's test.

Figure 2 shows the prevalence of hypertension in the four groups of participants categorized by enterotype and daily salt-intake. The prevalence of hypertension was significantly different (*p* < 0.05) for the two GM enterotype groups with low-salt intake (enterotype 1, 47%; enterotype 2, 27%). However, there was no significant difference (*p* = 0.83) in hypertension prevalence for the two GM enterotype groups with high-salt intake (enterotype 1, 50%; enterotype 2, 47%).

**Figure 3:**
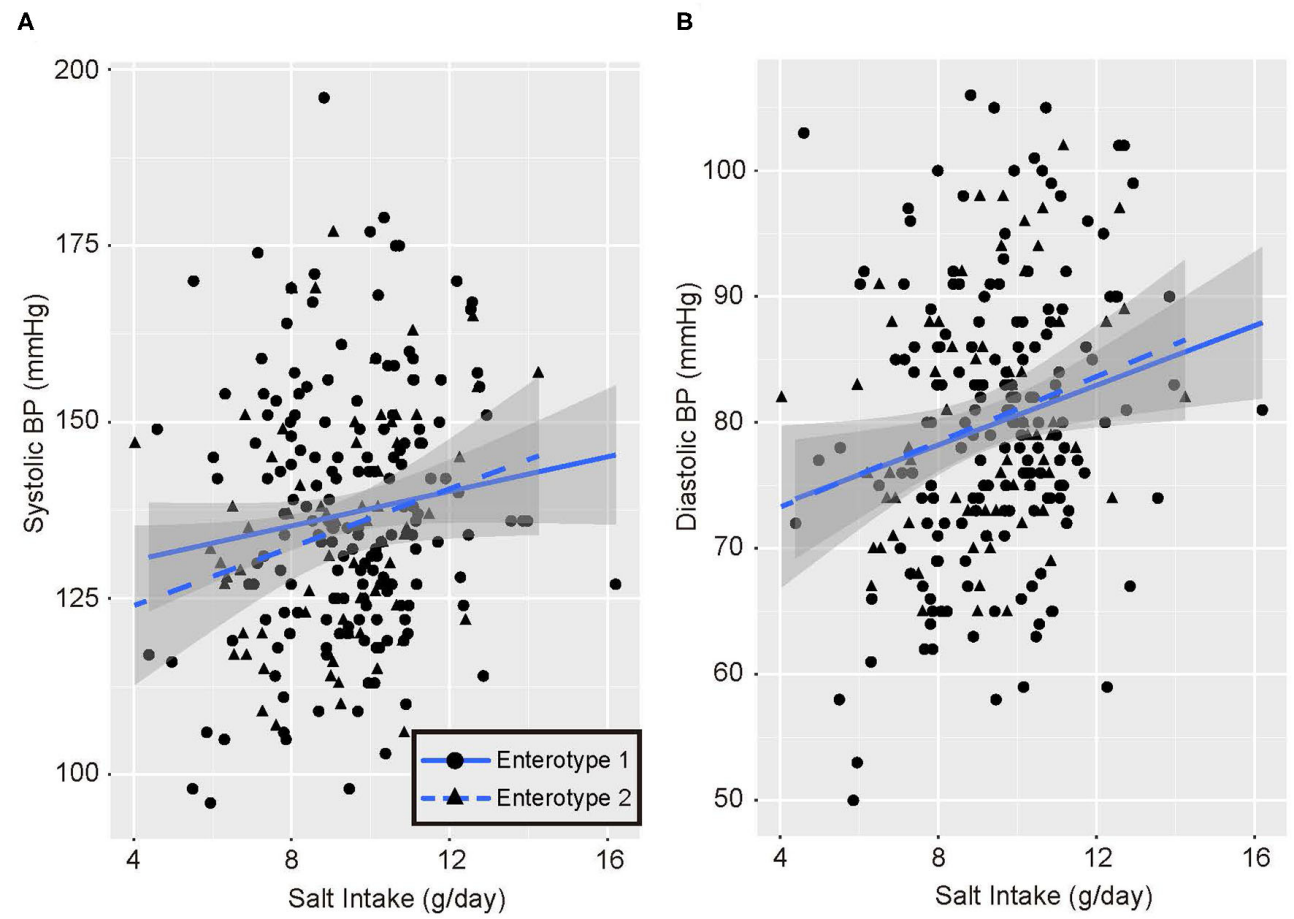
Blood pressure and salt intake scatter plots. **(A)** Systolic blood pressure (BP) and salt intake. **(B)** Diastolic blood pressure and salt intake. Black circles and solid blue line, enterotype 1; Black triangles and broken blue line, enterotype 2.

Univariate analysis and multiple logistic regression analysis adjusted for age, sex, and BMI revealed that predictive changes in GM enterotypes were associated with hypertension in the low-salt group (Supplemental Table 3). Scatter plots of systolic and diastolic BP vs. salt intake are shown in Figure 3. The slopes of the approximate straight lines were greater for enterotype 2 (SBP = 2.00, DBP = 1.30) than for enterotype 1 (SBP = 1.23, DBP = 1.18).

### Significant Intestinal Bacteria Genera

**Figure 4:**
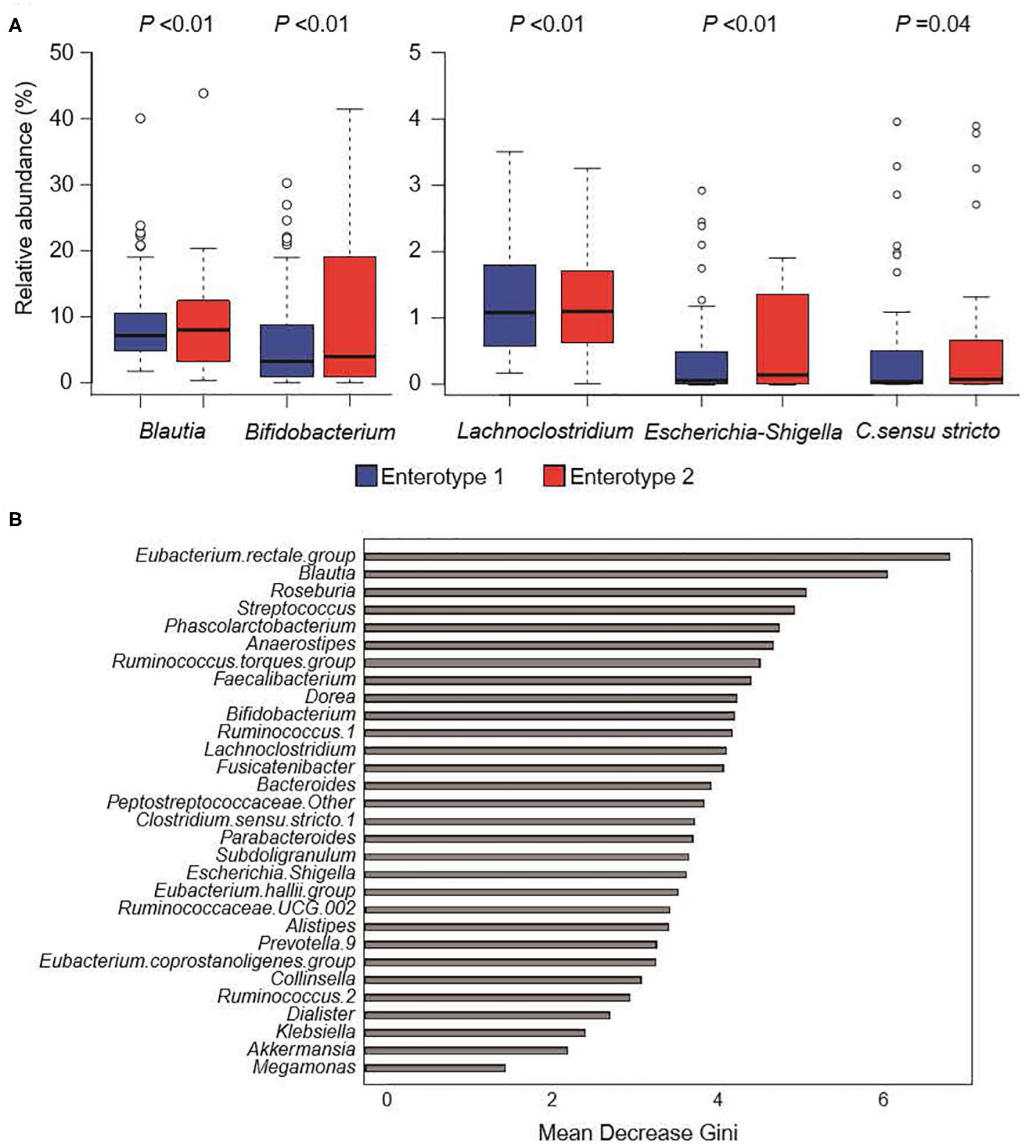
Gut microbiome composition in low-salt groups. **(A)** Relative abundance of gut microorganisms with significant differences between enterotype 1 and enterotype 2. *P*-values were calculated using DESeq2 differential gene expression analysis. **(B)** Random forest model using explanatory variables of gut microorganisms.

Figure 4 shows the GM compositions of the low-salt groups. The relative abundance of certain microorganisms was significantly different between enterotype 1 and enterotype 2 groups. For instance, the relative abundances of *Blautia, Bifidobacterium, Escherichia-Shigella, Lachnoclostridium*, and *Clostridium. sensu. stricto*. were significantly higher in the enterotype 2 groups. Feature importance ranking indicated that the *Eubacterium rectale* group and *Blautia* were the top two most discriminatory bacteria between enterotype 1 and enterotype 2 groups in the low-salt groups. Also, in high-salt group, *Bifidobacterium* and *Lachnoclostridium* differed significantly between enterotype 1 and enterotype 2 (*Bifidobacterium p* < 0.01, *Lachnoclostridium p* = 0.01), while*, Escherichia-Shigella, Clostridium sensu stricto.1, and Blautia* did not differ significantly *(Escherichia-Shigella p* = 0.09, *Clostridium sensu stricto.1 p* = 0.89, *Blautia p* = 0.99).

### The Renin-Angiotensin-Aldosterone System and Inflammatory Cytokines

Enterotype 1 and enterotype 2 were categorized using PCA. The PCA plot included age, sex, BMI, PRA, PAC, eGFR, IL-17a, GM-CSF, and TNF-α, past history of DM, and past history of HL. In the low-salt groups, there was a significant difference in these components between the enterotype 1 and enterotype 2 groups (PERMANOVA; *p* < 0.05). However, in the high-salt group and all-participants group, there were no significant differences between the enterotype 1 and enterotype 2 groups (Supplemental Figure 1A). Feature importance ranking revealed that GM-CSF, PRA, TNF-α, PAC, and IL-17a were the most discriminatory factors between the groups (Supplemental Figure 1B).

## Discussion

We defined two distinct enterotype groups based on the composition of fecal microbial communities in the general Japanese population. There was a significantly higher prevalence of hypertension in the enterotype 1 group with low-salt intake compared to the enterotype 2 group with low-salt intake. Six significant candidate bacterial genera were identified for classifying the two enterotypes with low-salt intake. *Blautia, Bifidobacterium, Escherichia-Shigella, Lachnoclostridium*, and *Clostridium sensu stricto* showed significant differences in their prevalence ratios for fecal bacteria genera and the *Eubacterium rectale* group and *Blautia* had higher feature importance rankings used to define the two enterotype groups with low-salt intake. Further, *Bifidobacterium* and *Lachnoclostridium* differed significantly between the two enterotype groups in both low-salt intake and high-salt intake.

Several previous studies have recently reported evidence that GM and the regulation of BP are linked and have described the potential mechanisms involved (14–16). For instance, the following candidate mechanisms for an association between GM and hypertension have been presented: (1) immunomodulatory function via GM (17, 36); (2) short-chain fatty acids (SCFA)-producing GM (37, 38); (3) trimethylamine N-oxide (TMAO) (39, 40); (4) glucocorticoid metabolism (41); and (5) epigenetic regulation (42–44).

First, GM demonstrates a multitude of physiological functions through the modulation of the host immune system (45). The subtle imbalance of GM composition may play a key role in the onset of hypertension. Wilck et al. showed that high-salt intake also drives autoimmunity by inducing T-helper (Th)17 cells, which then contribute to hypertension by depleting *Lactobacillus murinus* (17). They also demonstrated that a 14-d challenge of a high-salt diet increases BP and the number of circulating IL-17A^+^/TNF-α^+^/CD4^+^ T-cells while reducing fecal *Lactobacillus* species in humans (17). In our current investigation, there were few *Lactobacillus* and no significant differences in composition between enterotype 1 and enterotype 2. Therefore, *Lactobacillus* composition was not regarded as a contributor to the prevalence of hypertension in our population group. *Bifidobacterium* is reported to induce the development of regulatory T-cells (Treg) and Th17 cell compartments in the intestine and the secretion of IL-17 (46). This suggests that bacteria may play a role in balancing the development of Treg and Th17 cell compartments. This may induce an effector function such as secreting IL-17 or a regulatory action such as suppressing the activation of the immune system, depending on the environment and the nature of the stimuli, including high-salt intake (47).

Second, among the six candidate bacterial genera identified for classifying the two enterotypes with low-salt intake, *Blautia, Bifidobacterium, Lachnoclostridium*, and the *Eubacterium rectale* group are reported to be SCFA-producing bacteria. SCFAs are the major nutrients produced by bacterial fermentation with the three major SCFAs being acetate, propionate, and butyrate. Acetate was produced by all four of the bacteria genera. In addition, *Blautia obeum* is able to produce both propionate and butyrate (48) whereas *Eubacterium spp*. produces mainly butyrate. SCFAs are known to influence several aspects of host physiology, including the regulation of BP (49). SCFAs can influence host cells by interacting with host G protein-coupled receptor 41 (Gpr41) and olfactory receptor 78 (Olfr78). Intriguingly, Olfr78 null mice are hypotensive (37), whereas Gpr41 null mice are hypertensive (38). SCFAs are known to induce vasorelaxation (50, 51). Thus, SCFAs acting on Gpr41 in the vascular endothelium may help to set the vascular tone. These pathways may be physiologically important links between SCFAs and the control of host BP.

Third, TMAO is a small organic compound derived mainly from choline and is metabolized by the microbiota to produce trimethylamine (TMA). TMAO is a known predictor of prevalent cardiovascular diseases (CVDs) (52) and of future cardiovascular events in clinical cohorts (53). Ge et al. recently performed a systematic review and meta-analysis and found that subjects with high TMAO concentrations have a 12% greater risk of hypertension compared to those with low circulating TMAO concentrations (39). Furthermore, Martin et al. showed that TMAO prolongs the hypertensive effect of angiotensin II in rats (40). Therefore, TMAO may play a key mediator role in the development of hypertension via angiotensin II activation. However, there are currently few reports regarding the association of TMAO with the six genera of bacteria that we identified in the current study. Additional investigation is needed regarding this topic.

Fourth, other investigators have proposed an alternative relationship between hypertension-onset and GM system. Gut bacteria are involved in metabolizing the endogenous glucocorticoids corticosterone and cortisol (41), which are able to bind and activate mineralocorticoid receptors (MR), causing sodium retention, hypertension, and renal injury (54). 21-deoxycortisol is derived from 21-dehydroxylation of corticosterone or cortisol by intestinal bacteria and most notably the inhibition of 11β-hydroxysteroid dehydrogenase (11β-HSD) type 1 and type 2. Plasma levels of 21-deoxycortisol are elevated in humans with hypertension compared with that of normotensive controls (55).

Finally, recent studies have provided evidence for gut-derived effector molecules affecting host epigenetics as another mechanism of dynamic interactions between hosts and GM, including histone deacetylation. Diet, including salt intake, and GM can influence epigenetics (56–58). There are several reports on the relevance of Histone deacetylases (HDACs) to the development of hypertension (42–44, 59). In addition, SCFAs generated from SCFA-producing bacteria have histone deacetylase inhibitory activity and alter the expression of specific hypertension**-**related genes via conformational changes in the active site of HDAC, resulting in HDAC inactivation (60, 61). Pharmacological inhibition of HDACs is expected to be a practical novel therapeutic strategy for the treatment of hypertension.

While our current study provided important new details on the impact of the intestinal bacterial flora on hypertensive patients with high-salt intake, certain limitations should also be addressed. The study design was cross-sectional, and therefore, the causality of the relationships could not be assessed. Furthermore, although this study includes a relatively large amount of information on the Japanese general population, however, only small sample sizes were included for two of the four final groups, the low-salt/GM enterotype 2 and the high-salt/GM enterotype 2. Regarding the six candidate GM biomarkers we identified, further interventional studies are necessary using both animal- and human-based study designs to investigate the change in GM by salt-intake and the effect on BP by feces transplant, bacteria transplant, or prebiotics. Further, daily salt-intake was estimated by urine sodium levels and creatine ratios in spot urine samples, and not in the 24 h urine samples. However, Huang et al. reported that the estimated daily salt-intake by spot urine testing was unable to detect the differences in sodium excretion measured by 24 h urine samples (62). Spot urine-based methods may be enough to evaluate daily salt-intake.

In conclusion, the current study demonstrated that the prevalence of hypertension is associated with the constitution of fecal bacteria and salt intake, and six microbial genera related to hypertension prevalence were identified in subjects who had low-salt intake. This suggested that there may be individuals with a specific gut bacteria composition for which changing dietary habits to low salt would be ineffective in preventing hypertension. The specific gut bacteria composition may not mean large quantities of a specific bacteria but appropriate population ratio with some bacteria. Physicians could identify the specific GM composition of hypertensive patients on salt diets for whom control of blood pressure has been difficult and could provide them the appropriate advice regarding low salt diets. Furthermore, treatment for gut dysbiosis, such as the administration of probiotics or fecal microbiota transplantation, might be affected by the stability of BP control in patients with hypertension. Our findings indicate a new strategy for controlling BP and the development of hypertension through the restoration of GM homeostasis. However, further studies examining the prospective relationship between the microbiome and hypertension induced by high-salt intake and the detailed mechanisms are still needed.

## Data Availability Statement

The result in DDBJ (DRA009074) can be seen in NCBI at the following URL; https://www.ncbi.nlm.nih.gov/sra/?term=DRA009074.

## Ethics Statement

The studies involving human participants were reviewed and approved by the Ethics Committee for Human Studies at Kanazawa University Hospital. The patients/participants provided their written informed consent to participate in this study.

## Author Contributions

SN, SK, TY, and SO contributed to the study design and conducted the study. SN and SK wrote the manuscript. SN, SK, and HNam analyzed statistically. HTsuj, SM, AH, and HNak prepared the application to the ethics committee. MK, DA, TH, and YT collected the clinical data. SN, AY, and SO performed DNA extraction from stool samples and Next Generation Sequencing. HTsub and HS measured cytokine levels. SK, MD, and YT edited the manuscript. SN and SO are the guarantor of this work and, as such, had full access to all the data in the study and takes responsibility for the integrity of the data and the accuracy of the data analysis. All the authors have read the manuscript and have approved this submission. All authors contributed to the article and approved the submitted version.

## Conflict of Interest

The authors declare that the research was conducted in the absence of any commercial or financial relationships that could be construed as a potential conflict of interest.
