# Supplementary material for: Impact of Gut Microbiome on Hypertensive Patients With Low-Salt Intake: Shika Study Results
Source: Front Med (Lausanne). 2020 Sep 2;7:475. doi: 10.3389/fmed.2020.00475 (PMC7492604; doi:10.3389/fmed.2020.00475)
Supplement: Supplementary file 1 [file Data_Sheet_1.docx]

Supplementary Material

# Supplemental Figure Legends

**Supplemental Figure 1.** (A) Principal component analysis (PCA) plot by age, sex, body mass index (BMI), plasma renin activity (PRA), plasma aldosterone concentration (PAC), estimated glomerular filtration rates (eGFR), diabetes mellitus (DM) rate, hyperlipidemia (HL) rate, interleukin 17a (IL-17a), granulocyte-macrophage colony-stimulating factor (GM-CSF), and tumor necrosis factor alpha (TNF-α) levels. (B) Random forest model using explanatory variables of age, sex, BMI, PRA, PAC, eGFR, DM rate, HL rate, IL-17a, GM-CSF, and TNF-α.

# Supplementary Tables

**Supplemental Table 1.** Characteristics of the two study participant groups categorized based on gut microbiome clustering.

| Characteristics | Enterotype 1  (n = 172) | Enterotype 2  (n = 67) | p value |
| --- | --- | --- | --- |
| Age, y­­ | 63 ± 10 | 62 ± 10 | 0.43 |
| Female, % | 56.3 | 42.0 | 0.06 |
| BMI, kg/m^2^ | 23.4 ± 3.2 | 23.0 ± 3.0 | 0.37 |
| Hypertension, % | 26.0 | 41.8 | 0.46 |
| Hyperlipidemia, % | 26.6 | 25.3 | 1.00 |
| Diabetes mellitus, % | 7.7 | 11.9 | 0.47 |
| SBP, mmHg | 137 ± 18 | 135 ± 16 | 0.34 |
| DSP, mmHg | 80 ± 11 | 80 ± 9 | 0.78 |
| Salt intake, g/day | 9.5 ± 1.9 | 9.2 ± 1.9 | 0.24 |
| eGFR, mL/min/1.73 m^2^ | 67.9 ± 11.9 | 69.7 ± 12.6 | 0.38 |
| PRA, ng/mL/h | 2.3 ± 6.1 | 2.5 ± 4.4 | 0.98 |
| PAC, pg/mL | 139.2 ± 72.8 | 144.0 ± 59.9 | 0.37 |
| GM-CSF, pg/mL | 9.1 ± 14.6 | 5.6 ± 4.0 | 0.08 |
| IL17a, pg/mL | 2.1 ± 1.4 | 1.9 ± 1.1 | 0.10 |
| TNFα, pg/mL | 1.5 ± 0.7 | 1.3 ± 0.4 | 0.06 |

Abbreviations: BMI, body mass index; SBP, systolic blood pressure; DBP, diastolic blood pressure; eGFR, estimated glomerular ﬁltration rate; PAC, plasma aldosterone concentration; PRA, plasma renin activity; GM-CSF, granulocyte-macrophage colony-stimulating factor; IL-17a, interleukin-17a; TNFα, tumor necrosis factor alpha. P values were calculated using unpaired T-test or Fisher’s exact test.

**Supplemental Table 2.** p values derived for the comparison of participants information in four participant groups and between Enterotype 1 vs 2 in Low or High salt group (related to Table 1).

|  | p value | | |
| --- | --- | --- | --- |
| Characteristics | Four | Low salt | High salt |
|  | groups | Enterotype 1 vs 2 | Enterotype 1 vs 2 |
| Age, y | 0.64 | 0.31 | 0.93 |
| Female, % | 0.04 | 0.02 | 0.67 |
| BMI, kg/m^2^ | 0.08 | 0.80 | 0.47 |
| Hypertension, % | 0.14 | 0.04 | 0.83 |
| SBP, mmHg | 0.21 | 0.26 | 0.80 |
| DSP, mmHg | 0.34 | 0.81 | 0.63 |
| Salt intake, g/day | <0.01 | 0.58 | 0.69 |
| eGFR, mL/min/1.73m^2^ | 0.33 | 0.57 | 0.37 |
| PRA, ng/mL/hr | 0.4 | 0.39 | 0.24 |
| PAC, pg/mL | <0.01 | 0.23 | 0.55 |
| GM-CSF, pg/mL | 0.07 | 0.06 | 0.08 |
| IL17a, pg/mL | 0.07 | 0.28 | 0.15 |
| TNF-α, pg/mL | 0.20 | 0.40 | 0.06 |
| Antihypertensive, % | 0.10 | 0.06 | 0.24 |

Abbreviations: BMI, body mass index; SBP, systolic blood pressure; DBP, diastolic blood pressure; eGFR, estimated glomerular ﬁltration rate; PAC, plasma aldosterone concentration; PRA, plasma renin activity; GM-CSF, granulocyte-macrophage colony-stimulating factor; IL-17a, interleukin-17a; TNFα, tumor necrosis factor alpha. P values were calculated using unpaired ANCOVA test, T-test or Fisher’s exact test.

**Supplemental Table 3.** Univariate analysis and multiple logistic regression analysis adjusted for age, sex, and body mass index (BMI).

| Low salt group | | | | | High salt group | | | | |
| --- | --- | --- | --- | --- | --- | --- | --- | --- | --- |
| Value | Odds ratio | 95% CI | p value | Value | | Odds ratio | 95% CI | p value |  |
| Enterotype 1 or 2 | 0.42 | 0.18-0.98 | 0.04 | Enterotype 1 or 2 | | 0.90 | 0.4-2.06 | 0.79 |  |
| Value | Odds ratio | 95% CI | p value | Value | | Odds ratio | 95% CI | p value |  |
| Enterotype 1, or 2 | 0.39 | 0.15-0.99 | 0.05 | Enterotype 1 or 2 | | 0.91 | 0.39-2.15 | 0.83 |  |
| Age | 1.06 | 1.02-1.11 | 0.01 | Age | | 1.03 | 0.99-1.08 | 0.23 |  |
| Female | 0.54 | 0.23-1.27 | 0.15 | Female | | 0.64 | 0.3-1.37 | 0.24 |  |
| BMI | 1.22 | 1.07-1.39 | < 0.01 | BMI | | 1.12 | 0.98-1.3 | 0.12 |  |

Univariate analysis and multiple logistic regression analysis were performed to identify variables that predict hypertension. Multiple logistic regression analysis was used to adjust for age, sex, and BMI.

## Supplementary Figures


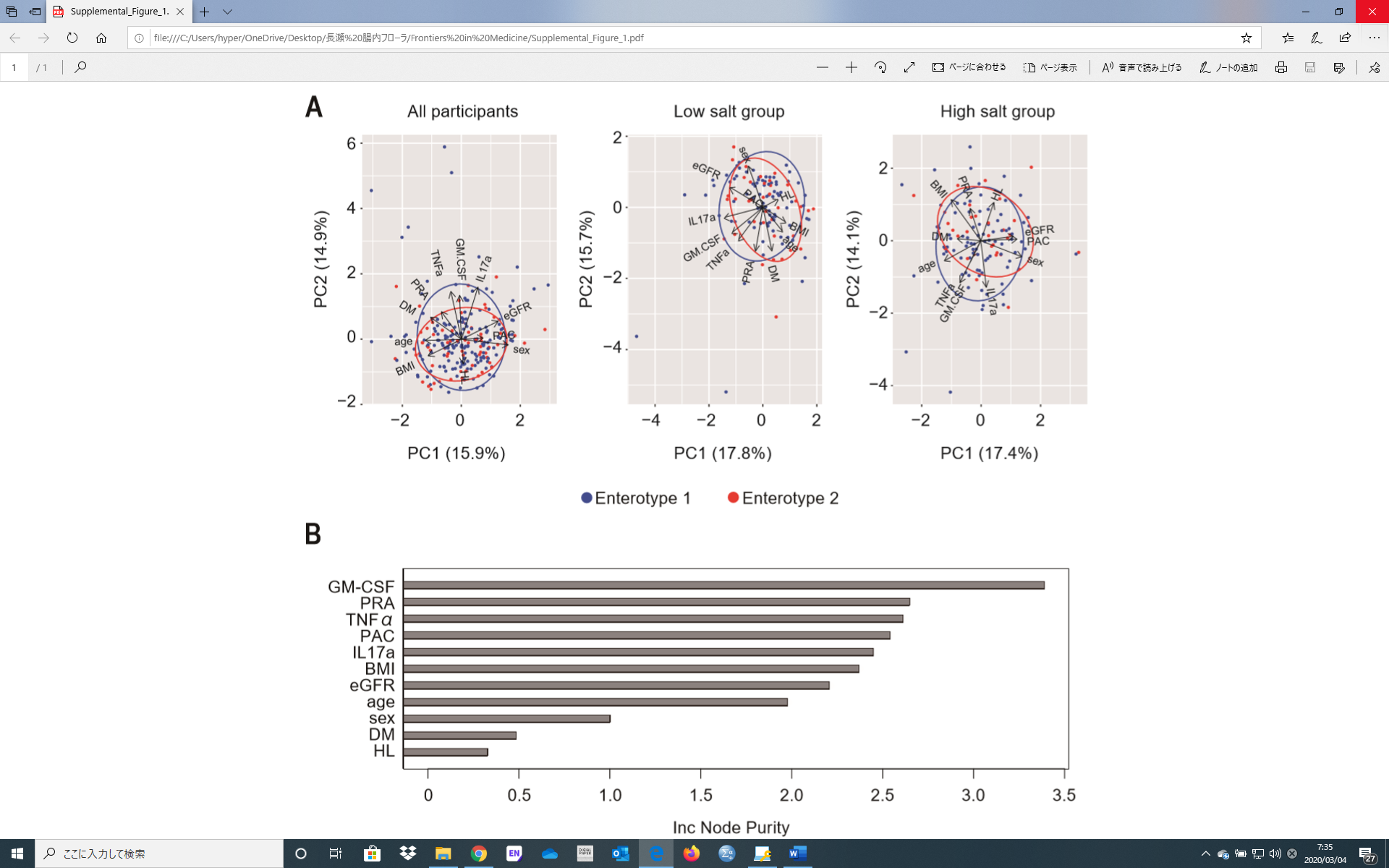


Supplemental Figure 1

**
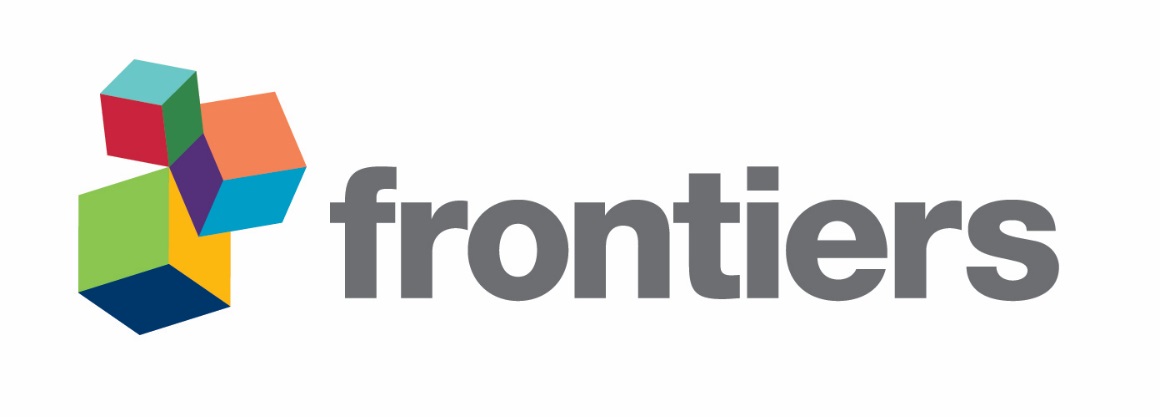
**
